# ATRvD1 Attenuates Renal Tubulointerstitial Injury Induced by Albumin Overload in Sepsis-Surviving Mice

**DOI:** 10.3390/ijms222111634

**Published:** 2021-10-27

**Authors:** José Bruno N. F. Silva, Thayanne B. B. Calcia, Cyntia P. Silva, Rafael F. Guilherme, Fernando Almeida-Souza, Felipe S. Lemos, Kátia S. Calabrese, Celso Caruso-Neves, Josiane S. Neves, Claudia F. Benjamim

**Affiliations:** 1 Institute of Microbiology Paulo de Góes (IMPG), Federal University of Rio de Janeiro (UFRJ), Rio de Janeiro 21941-902, Brazil; nunes.brj@mail.uft.edu.br (J.B.N.F.S.); rafael_guilherme@hotmail.com (R.F.G.); 2 Institute of Biophysics Carlos Chagas Filho (IBCCF), Federal University of Rio de Janeiro (UFRJ), Rio de Janeiro 21941-902, Brazil; thayannecalcia@gmail.com (T.B.B.C.); caruso@biof.ufrj.br (C.C.-N.); 3 Institute of Biomedical Sciences (ICB), Federal University of Rio de Janeiro (UFRJ), Rio de Janeiro 21941-902, Brazil; cyntiapecli@hotmail.com (C.P.S.); simoeslemos.f@gmail.com (F.S.L.); jneves@icb.ufrj.br (J.S.N.); 4 Laboratory of Immunomodulation and Protozoology, Oswaldo Cruz Institute (IOC), Fiocruz, Rio de Janeiro 21040-900, Brazil; fernandoalsouza@gmail.com (F.A.-S.); calabrese@ioc.fiocruz.br (K.S.C.); 5 Postgraduate in Animal Science, State University of Maranhão, São Luís 65055-310, Brazil

**Keywords:** sepsis, renal tubulointerstitial injury, resolvin, ATRvD1, inflammation, kidney

## Abstract

Novel strategies for the prevention and treatment of sepsis-associated acute kidney injury and its long-term outcomes have been required and remain a challenge in critical care medicine. Therapeutic strategies using lipid mediators, such as aspirin-triggered resolvin D1 (ATRvD1), can contribute to the resolution of acute and chronic inflammation. In this study, we examined the potential effect of ATRvD1 on long-term kidney dysfunction after severe sepsis. Fifteen days after cecal ligation and puncture (CLP), sepsis-surviving BALB/c mice were subjected to a tubulointerstitial injury through intraperitoneal injections of bovine serum albumin (BSA) for 7 days, called the subclinical acute kidney injury (subAKI) animal model. ATRvD1 treatment was performed right before BSA injections. On day 22 after CLP, the urinary protein/creatinine ratio (UPC), histologic parameters, fibrosis, cellular infiltration, apoptosis, inflammatory markers levels, and mRNA expression were determined. ATRvD1 treatment mitigated tubulointerstitial injury by reducing proteinuria excretion, the UPC ratio, the glomerular cell number, and extracellular matrix deposition. Pro-fibrotic markers, such as transforming growth factor β (TGFβ), type 3 collagen, and metalloproteinase (MMP)-3 and -9 were reduced after ATRvD1 administration. Post-septic mice treated with ATRvD1 were protected from the recruitment of IBA1^+^ cells. The interleukin-1β (IL-1β) levels were increased in the subAKI animal model, being attenuated by ATRvD1. Tumor necrosis factor-α (TNF-α), IL-10, and IL-4 mRNA expression were increased in the kidney of BSA-challenged post-septic mice, and it was also reduced after ATRvD1. These results suggest that ATRvD1 protects the kidney against a second insult such as BSA-induced tubulointerstitial injury and fibrosis by suppressing inflammatory and pro-fibrotic mediators in renal dysfunction after sepsis.

## 1. Introduction

Sepsis is a life-threatening condition caused by a systemic inflammatory response to infection, resulting in multisystem organ failure and death [1]. The pathophysiology of sepsis involves innate-immune cell activation triggered by pattern recognition receptors, which recognize pathogen-associated molecular patterns and endogenous damage-associated molecular patterns alongside profound circulating B and T cells, which release excessive amounts of pro-inflammatory and anti-inflammatory cytokines. This cytokine storm induces endothelial and microvascular dysfunction, tissue damage, and apoptosis, being closely associated with multiple organ failure [2].

Prevention, early recognition of at-risk patients, and efficient supportive care have all contributed to a decline in short-term mortality among septic patients [3,4,5,6]; however, long-term adverse outcomes are still evident after sepsis with impaired immune response in vital organs [7,8]. Following hospital discharge, one in five sepsis survivors is re-hospitalized due to infections or other acquired comorbidities [9]. In this regard, acute kidney injury (AKI) is reported in 50% of sepsis patients with an ICU mortality rate of 41% [10]; additionally, AKI is one of the causes of readmissions in septic patients who were discharged. Thus, kidney dysfunction is an important acute and long-term outcome with a negative impact on quality of life [11]. To attenuate the long-term implications of renal failure, pharmacological strategies that promote kidney recovery are required.

While kidney dysfunction is strongly associated with mortality and other adverse outcomes in sepsis survivors, the inflammatory and immune pathways that lead to this phenomenon are not well-elucidated [12]. In brief, kidney damage triggers the release of tumor necrosis factor-α (TNF-α), interleukin (IL)-1β, IL-6, and IL-8, which stimulate prolonged activation of inflammatory cells. Simultaneously, regenerative mechanisms occur via infiltrative and tissue resident cells, which secret pro-fibrotic factors, including transforming growth factor-β (TGFβ) and extracellular matrix (ECM). As it is known, hypoxia, oxidative stress, and autophagy are involved in AKI. This persistent inflammation coupled with an uncontrolled repair process causes an excessive ECM deposition, leading to the formation of interstitial fibrosis, which is the signature of progressive loss of function [13,14]. In addition, sepsis-related AKI negatively impacts distant organs such as the brain, gut, heart, liver, and lungs [15].

By considering that inflammation is one of the major components of organ dysfunctions in sepsis, we previously investigated whether a second-hit challenge exacerbates inflammation and tissue injury using a subclinical acute kidney injury (subAKI) model. This model is characterized by an acute tubule-interstitial injury without changes in glomerular function and structure. Then, we verified renal damage in sepsis-surviving mice, being characterized by higher levels of the urinary protein and creatinine (UPCr) ratio, which was aggravated when post-septic mice were challenged with intraperitoneal bovine serum albumin (BSA) [16].

Specialized pro-resolving mediators (SPMs) derived from polyunsaturated fatty acids (PUFAs) potentially play an important role as pro-resolution and anti-inflammatory derivatives on inflammation. D- and E-series resolvins (RvD and RvE, respectively) are some of those mediators, and they are biosynthesized by docosahexaenoic acid (DHA) and eicosapentenoic acid (EPA), respectively, via 15/5 lipoxygenase (LOX) or by aspirin-acetylated COX-2, which generates RvD epimers, called aspirin-triggered RvD (ATRvD), and 18*S*-RvE [17].

The potential role of SPMs in the resolution of sepsis has been explored. While widespread systemic inflammation induced higher levels of leukotriene B_4_ and prostaglandin E_2_ in sepsis non-survivors, increased levels of RvE1 and RvD5 were associated with survival subjects [18]. The treatment of polymicrobial sepsis in mice with RvD1 and RvD2 reduced the inflammatory cytokine storm and neutrophil migration, enhanced bacterial clearance, and improved the survival rate [19,20]. In addition, Zhuo et al. demonstrated that RvD1 attenuated lung injury during systemic infection using the murine cecal ligation and puncture (CLP) model [21]. Additionally, ATRvD1, a more potent and stable mediator than RvD1 [22], was able to reduce endotoxemia-induced AKI and to limit neutrophilic infiltration [23,24].

Based on the reported effects of RvD series, we postulated that ATRvD1 could attenuate long-term kidney dysfunction induced by a second insult after severe sepsis. Therefore, we evaluated the protective role of ATRvD1 on tubulointerstitial injury induced by BSA overload in a sepsis-surviving mouse model.

## 2. Results

### 2.1. ATRvD1 Treatment Ameliorated BSA-Induced Kidney Tubulointerstitial Injury in Post-Septic Mice

**Figure 1:**
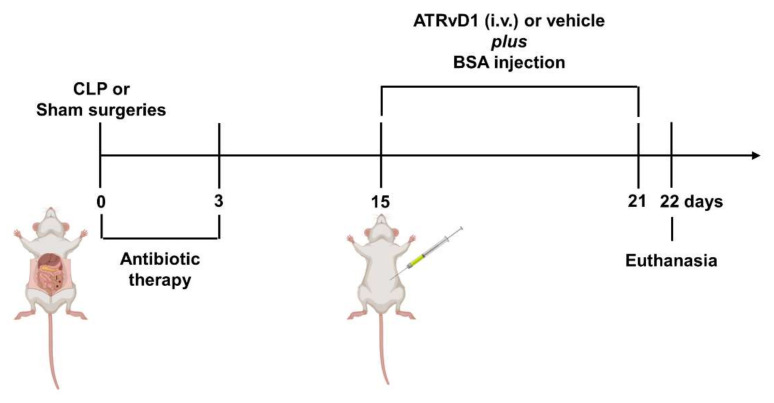
SubAKI model in sepsis-surviving mice and ATRvD1 treatment. Polymicrobial sepsis was induced by CLP model. Following anesthesia, the cecum of septic animals was punctured twice (21G) before returning to the abdominal cavity. Sham-operated mice (Sham) were subjected to an identical laparotomy but without CLP. All mice were i.p. treated with ertapenem antibiotic (75 mg/kg) for three consecutive days. At day 15 after surgery, sepsis-surviving and Sham animals were divided into groups and received 10 g/kg BSA (i.p.) for seven consecutive days. Mice received 5 μg/kg ATRvD1 (0.1% ethanol, i.v.) at day 15 post-surgery followed by 0.5 μg/kg (0.01% ethanol) 20 min prior to BSA challenge for six days. The control groups received 0.1 or 0.01% ethanol (vehicle, i.v.) at the same days and volumes (100 μL) as the ATRvD1-treated groups. At day 22, the mice were anesthetized, and blood samples were collected by cardiac puncture. Kidneys were then immediately removed postmortem. (n = 3–10 for each experimental group.)

Based on our previous data, sepsis-surviving mice already presented tubulointerstitial injury at days 7 and 14, which was aggravated after BSA challenge [16]. Along these lines, renal morphology was analyzed to determine whether ATRvD1 treatment was able to attenuate BSA-induced kidney insult after CLP using a subAKI animal model (Figure 1). 

**Figure 2:**
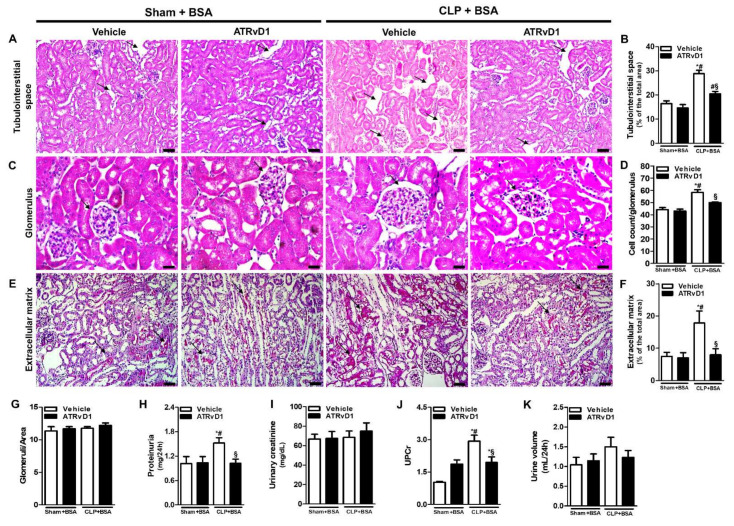
ATRvD1 treatment ameliorates BSA-induced kidney tubulointerstitial injury in post-septic mice. (**A**,**B**) Tubulointerstitial space (arrows) and quantitative analysis were assessed in tissue sections after hematoxylin and eosin staining (magnification 20×, scale bar = 50 μm); (**C**,**D**) the glomerular cell numbers (arrows) were estimated in kidney tissue sections after hematoxylin and eosin staining (magnification 40×, scale bar = 20 μm). (**E**,**F**) Extracellular matrix deposition (arrows) and quantitative analysis were assessed in tissue sections after Sirius Red staining (magnification 20×, scale bar = 50 μm). Images are representative of each group (n = 3–5 for each experimental group); (**G**) glomeruli number was assessed in tissue sections after hematoxylin and eosin staining; (**H**–**K**) renal function was evaluated by proteinuria, urinary creatinine, urine volume, and urinary protein/creatinine ratio (UPC); (K) in the different experimental groups (n = 3–10 for each experimental group). Graphics represent means ± SE. * *p* < 0.05 compared with the non-treated Sham+BSA group; ^#^ *p* < 0.05 compared with Sham+BSA+ATRvD1; ^§^ *p* < 0.05 compared with non-treated CLP+BSA.

On day 22 post-surgery, non-treated CLP+BSA mice presented an increased kidney tubulointerstitial space (Figure 2A,B), glomerular cell number (Figure 2C,D), and a higher ECM deposition (Figure 2E,F), compared with Sham+BSA mice. The administration of ATRvD1 attenuated those histomorphology changes (Figure 2A,B,E,F). The same feature was observed for glomerular cell number (Figure 2C,D). Though, a similar number of glomeruli was found in all groups (Figure 2G). In addition, there were no differences in renal morphology between the Sham+BSA and Sham+BSA+ATRvD1 mice.

We then measured the proteinuria, urinary creatinine, and urinary protein/creatinine ratio (UPCr). As shown in Figure 2H, the proteinuria levels were increased in the CLP+BSA group, while ATRvD1 treatment showed a reduction in this parameter. However, the urinary creatinine levels remain similar between the groups (Figure 2I). Regarding the UPC ratio in 24 h, the results obtained indicate that ATRvD1 treatment significantly reduced this parameter compared with the CLP+BSA group (Figure 2J). There was no difference in urine output among all groups (Figure 2K). 

### 2.2. ATRvD1 Treatment Attenuated Collagen Deposition in BSA-Induced Tubulointerstitial Injury in Post-Septic Mice

**Figure 3:**
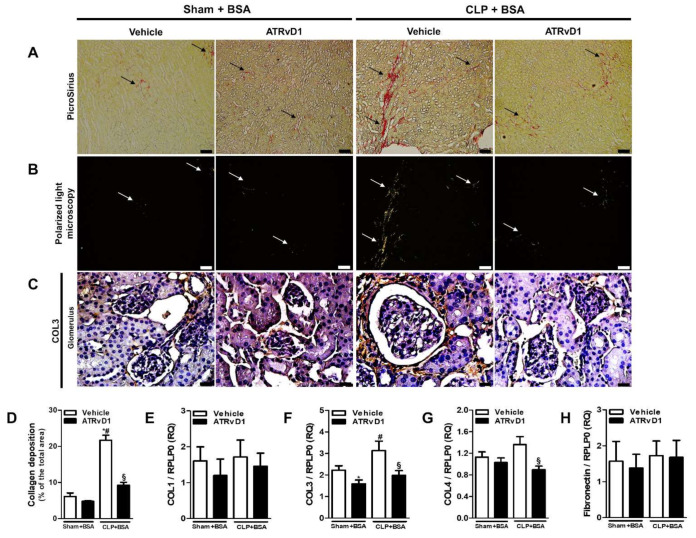
ATRvD1 treatment attenuates collagen deposition in BSA-induced injury. (**A**) Representative images of renal tissue sections after PicroSirius staining (magnification 10×, scale bar = 100 μm), arrows indicate collagen deposition; (**B**) polarized light micrographs of the renal tissue (magnification 10×, scale bar = 100 μm), arrows indicate collagen deposition; and (**C**) representative images of glomerular areas of the renal tissue after immunohistochemical staining for COL3 (magnification 40×, scale bar = 20 μm). Images are representative of each group (n = 3–10). (**D**) Quantitative analysis of collagen fibers deposition; (**E**–**H**) relative mRNA expression of COL1, COL3, COL4, and Fibronectin with RLP0 as an endogenous control. (n = 3–10 for each experimental group). Graphs represent means ± SE. * *p* < 0.05 compared with the non-treated Sham+BSA group; ^#^ *p* < 0.05 compared with Sham+BSA+ATRvD1; ^§^ *p* < 0.05 compared with non-treated CLP+BSA.

We investigated the kidney collagen and fibronectin deposition in order to evaluate the anti-fibrotic effect of ATRvD1 treatment. Figure 3A represents photomicrographs of histological slides of kidney tissue specimens stained with Picrosirius Red. An increased collagen deposition was observed in the CLP+BSA mice, whereas ATRvD1 treatment avoided it (Figure 3A,D). We then attempted to identify the type of collagen fibers in the renal tissue. Under polarized light, renal tissue from CLP+BSA mice contained predominantly green collagen fibers compared with that of CLP+BSA+ATRvD1 mice (Figure 3B), suggesting COL3 presence. Corroborating this, we demonstrated more COL3 in the glomerular zones of CLP+BSA mice by immunohistochemical analysis compared with that of CLP+BSA+ATRvD1 mice (Figure 3C). Additionally, COL1 deposition was undetectable in the renal tissue. Furthermore, the upregulated gene expression of COL3 and COL4 was inhibited by ATRvD1 treatment, while a similar gene expression was detected for COL1 and fibronectin (Figure 3E–H).

**Figure 4:**
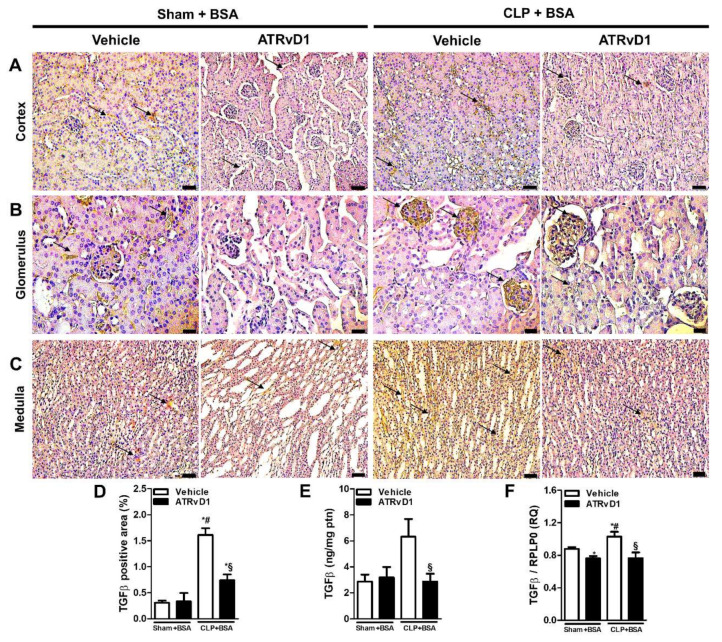
ATRvD1 treatment attenuates TGFβ expression in BSA-induced tubulointerstitial injury in post-septic mice. (**A**–**C**) Representative images (n = 3) of cortical (magnification 20×, scale bar = 50 μm), glomerular (magnification 40×, scale bar = 20 μm), and medullary (magnification 20×, scale bar = 50 μm) areas of the renal tissue after immunohistochemical staining for TGFβ (arrows). (**D**) Quantitative analysis of TGFβ positive area in renal tissue. (**E**) Renal TGFβ levels were measured using ELISA. (**F**) Relative mRNA expression of TGFβ with RLP0 as an endogenous control (n = 3–10 for each experimental group). Graphs represent means ± SE. * *p* < 0.05 compared with the non-treated Sham+BSA group; ^#^ *p* < 0.05 compared with Sham+BSA+ATRvD1; ^§^ *p* < 0.05 compared with non-treated CLP+BSA.

In response to injury, TGFβ is known to play a key role in fibrotic progression of the kidney. TGFβ was evaluated in the kidney tissue specimens using the immunohistochemistry, ELISA, and RT-PCR methods. The CLP+BSA group exhibited increased TGFβ expression in the cortical, medullar, and glomerular (Figure 4A–D) areas, compared with the Sham+BSA groups. All parameters were reduced to control levels by ATRvD1 treatment (Figure 4A–D). Corroborating this, the TGFβ protein levels and mRNA expression were elevated in the kidney tissues of the CLP+BSA group, and it was downregulated by ATRvD1 treatment (Figure 4E,F).

### 2.3. ATRvD1 Treatment Attenuated Inflammatory Markers in subAKI

**Figure 5:**
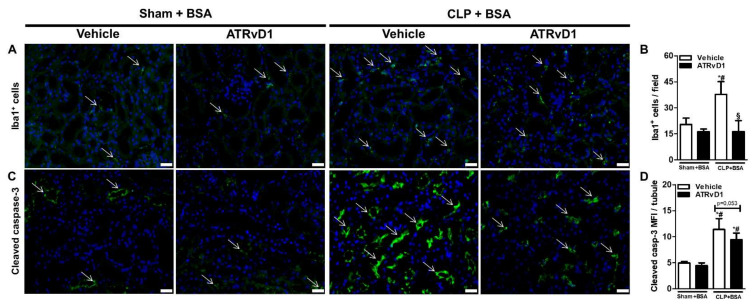
ATRvD1 treatment attenuates inflammatory markers in subclinical acute kidney injury. (**A**) Representative images of tissue renal sections after immunofluorescence staining for Iba1^+^ cells (green, arrows) and DAPI (blue) (magnification 40×, scale bar = 50 μm) as described in the Material and Methods section. (**B**) Quantitative analysis of Iba1^+^ cells in renal tissue. (**C**) Renal tissue immunolabelled for cleaved caspase-3 (green, arrows) and DAPI (blue) (magnification 40×, scale bar = 50 μm). (**D**) Mean fluorescence intensity (MFI) of cleaved caspase-3 (casp-3) in renal tubular cells. (n = 3 for each experimental group). Graphs represent means ± SE. * *p* < 0.05 compared with the non-treated Sham+BSA group; ^#^ *p* < 0.05 compared with Sham+BSA+ATRvD1; ^§^ *p* < 0.05 compared with non-treated CLP+BSA.

Previous studies have demonstrated the accumulation of macrophages and increased apoptosis in renal tissue caused by BSA challenge [16,25]. Therefore, we investigated whether ATRvD1 was able to reduce the number of ionized calcium-binding adopter molecule 1 (Iba1^+^) cells and the amount of apoptosis in the renal cortex. Immunofluorescence staining of kidneys revealed that CLP+BSA mice treated with ATRvD1 exhibited a marked decrease in the number of Iba1^+^ cells (Figure 5A,B) and reduced expression of cleaved caspase-3 (casp-3) in renal tissue (*p* = 0.053) (Figure 5C,D).

### 2.4. ATRvD1 Treatment Modulated MMP and Cytokine Production in BSA-Induced Tubulointerstitial Injury in Post-Septic Mice

**Figure 6:**
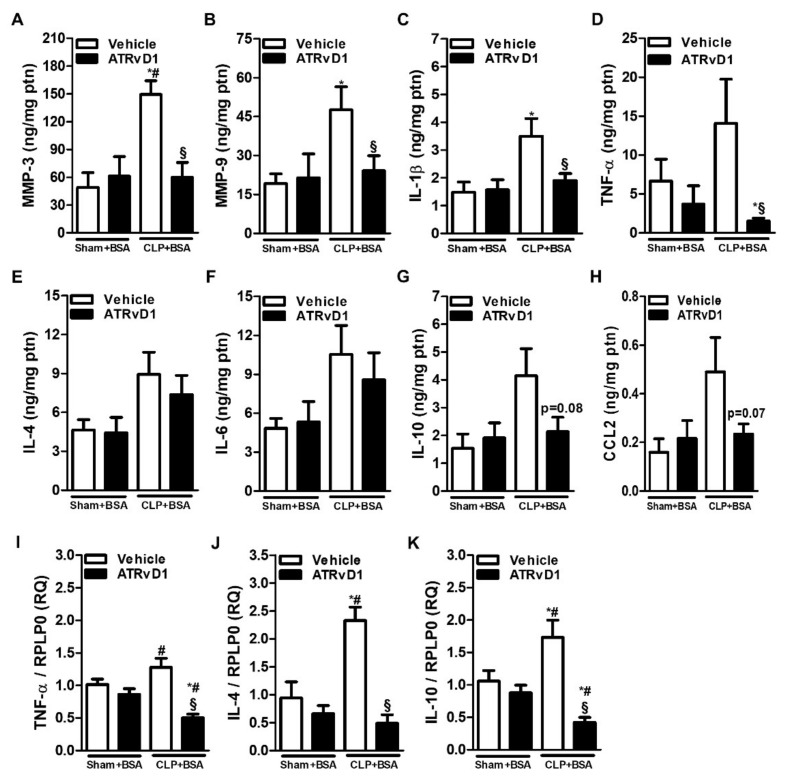
Matrix metalloproteinases and cytokine production are modulated by ATRvD1. (**A**–**H**) MMP-3, MMP-9, IL-1β, TNF-α, IL-4, IL-6, IL-10, and CCL2 levels were determined by ELISA. (**I**–**K**) Relative mRNA expression of TNF-α, IL-4, and IL-10 with RLP0 as an endogenous control (n = 3–10 for each experimental group). Graphs represent means ± SE. * *p* < 0.05 compared with the non-treated Sham+BSA group; ^#^ *p* < 0.05 compared with Sham+BSA+ATRvD1; ^§^ *p* < 0.05 compared with non-treated CLP+BSA.

MMP-3 and MMP-9 perform pro-fibrotic roles by activating latent TGFβ [26]. Therefore, the MMP-3 and MMP-9 levels were elevated in the CLP+BSA group compared with those of Sham groups. Interestingly, ATRvD1 administration was able to be reduced to the basal levels observed in the control groups (Figure 6A,B). 

Pro-inflammatory and anti-inflammatory cytokine release was previously reported in the BSA-induced tubulointerstitial injury of post-septic mice [16]. IL-1β levels was increased in the CLP+BSA group compared with Sham groups, and it was abolished by ATRvD1 treatment (Figure 6C). Despite the fact that no statistical differences were observed between the CLP+BSA and Sham groups with regard to TNF-α, IL-4, IL-6, IL-10, and CCL2 protein levels, greater amounts of these cytokines in the CLP+BSA group were observed (Figure 6D–H). Still, ATRvD1 therapy was able to reduce TNF-α, IL-10, and CCL2 levels but not IL-4 and IL-6 levels (Figure 6D–H). In addition, the upregulated mRNA expressions of TNF-α, IL-4, and IL-10 in the CLP+BSA group were significantly reduced by ATRvD1 therapy (Figure 6I–K). 

## 3. Discussion

The long-term dysfunction outcomes in sepsis-survivors are potentially linked to progressive impairment of quality of life and increased mortality [27]. Therapeutic strategies for persistent kidney dysfunction and increased pre-existing renal disorders after sepsis are limited. Our group has been engaged in investigating the recovery of homeostasis following long-term outcomes after sepsis [28].

Focusing on renal treatment for sepsis survivors, we used a subAKI model to investigate the potential effect of ATRvD1 on kidney dysfunction in sepsis-surviving mice. In our previous data, BSA challenge leads to inflammatory cytokine release and immune dysfunction in the cortical and medullary areas, which aggravates tubule damage and interstitial inflammation triggered by sepsis [16]. In addition, alterations in the albumin reabsorption machinery and changes in collagen deposition occur during this process [29]. We report here that ATRvD1 treatment reduced proteinuria excretion, the UPC ratio, the glomerular cell number, and ECM deposition. ATRvD1 also attenuated inflammatory cytokines release and their mRNA expression as well as cellular infiltration into kidney tissue of sepsis-surviving mice.

Tubular damage and renal fibrosis have been reported in the AKI experimental model [30], including the albumin overload strategy [25]. While Portella et al. did not relate fibrosis and tubulointerstitial space alterations in situ [16], we verified that tubulointerstitial damage and renal fibrosis progression were reverted with ATRvD1 treatment. Our findings are supported by the ability of RvD and RvE in reducing interstitial fibrosis and myofibroblast proliferation in the kidney [31,32]. In addition to reducing kidney fibrosis, both omega-3 supplementation (which increases endogenous renal levels of SPMs) and RvD1 attenuated tubulointerstitial injury and proteinuria [33,34]. ATRvD1 also ameliorated endotoxemic renal failure in mice [20,35].

COL1, COL3, and COL4 are constitutively expressed in normal kidney; however, during inflammation and fibrosis, an imbalance between formation and degradation of ECM occurs [36,37]. In the early phase of tissue repair, COL3 deposition is increased, followed by COL1 during renal fibrotic progression [38]. In addition, the fibrillogenic COL1 process involves COL3 participation, which regulates the collagen fibril diameters [39]. Particularly for COL4, its overexpression is associated with glomerular diseases [40,41]. Our data provide the first evidence that ATRvD1 administration reduces total collagen accumulation, and COL3 secretion and gene expression in sepsis-surviving mice challenged with a second hit in the subAKI model. However, COL1 deposition was not detected in renal tissue and its mRNA expression was unchanged. Although we did not find higher COL4 expressions in this model either, sepsis-surviving mice treated with ATRvD1 presented lower COL4 mRNA expression, showing the ability of ATRvD1 in modulating its synthesis in the renal tissue. Corroborating this, in a unilateral ureteric obstruction (UUO) model, RvD1 and RvE1 inhibited COL1, COL3, and COL4 accumulation [32,42]. 

During persistent tubulointerstitial injury, tubular cells drive inflammation and fibrosis and can acquire pro-fibrotic phenotype. These events are related to ECM replacement of cell loss, collagen turnover, and macrophage infiltration, which elicited chronic kidney disease, being evident in the renal failure [43]. We believe that abnormal COL1 and COL4 content may contribute to the destruction of the renal architecture and consequently impairs the function in advanced renal lesions, being not associated with renal fibrosis at the time evaluated in our model.

TGFβ activates pro-fibrotic pathways, leading to ECM deposition. BSA overload induces high TGFβ levels, which correlates with the renal damage [25,29]. In our study, ATRvD1 was able to counteract elevated TGFβ levels and mRNA expression stimulated by BSA overload. In line with this mechanism, Zheng et al. demonstrated that RvD1 acts by reducing TGFβ-induced collagen production [44]. Regarding decreased TGFβ in our study, RvD1 and ATRvD1 are potent regulators of TGFβ expression and production in acute and chronic inflammation [45,46,47]. In summary, these results strongly suggest that ATRvD1 plays pro-resolving actions in reducing pro-fibrotic mediators, including ECM secretion, through TGFβ suppression.

MMP-9, activated by MMP-3, may be required for the release of active TGFβ [48,49]. MMPs can be seen constitutively in tubules and glomerulus; however, its expression is increased in AKI [37]. In our model, a possible mechanism for reducing TGFβ is the attenuation of MMP-3 and MMP-9 triggered by ATRvD1 treatment. Similarly, Posso et al. demonstrated that ATRvD1 was able to downregulate MMP-3 expression in an emphysema model [50]. However, the effects of resolvins in the regulation of MMPs in renal disorders remain to be clarified.

Post-septic mice challenged with BSA present an immune dysfunction and inflammatory processes [16]. Consistent with those findings, we observed that ATRvD1 treatment attenuated inflammatory cytokine release as well as mRNA expression. There is evidence that RvD1 and RvD2 regulate pro- and anti-inflammatory cytokines in sepsis models [19,20,21]. The contribution of TNF-α to renal damage was confirmed using TNFR1^−/−^ in endotoxemia, which prevents inflammation and apoptosis [51]. In addition, the increased expression of casp-3 cleavage has been detected in endotoxin- and CLP-induced AKI [52,53], along with higher renal IL-1β levels in cisplatin-associated AKI [54]. It is known that IL-1β activates nuclear factor kappa B pathway, playing an important role in the transcription of pro-inflammatory cytokines. Furthermore, based on evidence that IL-1β induces cellular recruitment, proteinuria, and increased CCL2 and TNF-α levels in kidney [55,56], our results demonstrated that IL-1β attenuation by ATRvD1 treatment can be associated with the decreased Iba1^+^ cell recruitment, lower TNF-α levels, and attenuation of cleaved casp-3 in renal tubular cells (*p* = 0.053).

In renal cells, transient or persistent abnormal IL-10 expression is linked to TGFβ stimulus to promote fibrosis and progression of kidney disorders [57]. Our results indicate that the IL-10 reduction could be correlated to the TGF-β mitigation triggered by ATRvD1 treatment. Regarding IL-4, its upregulation induces fibrosis and tissue lesion aggravation and can protect tubular cell from damage [29]. Recently, Peruchetti et al. reported that IL-4 ameliorates tubulointerstitial injury induced by albumin overload [29]. However, IL-4 leads to the suppression of cell-mediated immunity and death in the CLP model [58]. In addition, Song et al. demonstrated that IL-4 contributes to lung function impairment of CLP surviving mice submitted to a second-hit infection with *Pseudomonas aeruginosa* and its neutralization was associated with pulmonary bacterial clearance [59]. In our study, sham and CLP mice secreted similar amounts of IL-4, but ATRvD1 was able to reduce its mRNA expression in sepsis-surviving mice. Once we developed a first- and second-hit model characterized by persistent inflammation, the exact role by which IL-4 participates in the resolution of this process remains to be further established.

Our results suggest that the role of TGFβ in renal fibrosis could be addressed through Smad proteins, in which Smad3 induces ECM synthesis [60], but its deletion inhibits tubulointerstitial fibrosis [61]. In this way, RvD1 attenuated renal fibrosis through the inhibition of Smad2 linker phosphorylation in the UUO model [42]. In the other hand, ATRvD1 activates Smad7, a key mediator that inhibits the TGFβ family and Smad2/3 signaling [62]. Other possible mechanisms may be addressed to ALX/FPR2, one of the known RvD receptors. Interestingly, the RvD1-ALX/FPR2 axis inhibited TNF-α receptor signaling and renal fibrosis [34]. Zhao et al. described that the activation of ALX/FPR2 in endotoxemia-induced AKI attenuated the inflammatory response [35]. In addition, mesangial cells proliferation can be inhibited through ALX/FPR2 mediation [63]. Along these lines, the inhibition of ALX/FPR2 axis aggravated sepsis-induced kidney damage [64]. These reports allow us to speculate that ATRvD1 exerts anti-inflammatory and pro-resolving actions by attenuating Smad 2/3 and by activating Smad7 signaling, thereby suppressing TGFβ activation, MMP3 and MMP9 release, collagen deposition, cytokine production, cell recruitment, apoptosis, as well as tubule interstitial damage triggered by ATRvD1-ALX/FPR2 axis. Nevertheless, our findings require further investigation using our experimental model to explore the mechanisms of ATRvD1 restoration of kidney function. 

## 4. Materials and Methods

### 4.1. Animals

Male and female BALB/c mice (8–12 weeks old; 18–25 g; 3–10 animals for each experimental group) were used in the study. The animals were obtained from the Brazilian National Institute of Cancer (Rio de Janeiro, Brazil) and Multidisciplinary Center for Biological Investigation (UNICAMP, São Paulo, Brazil). The mice were housed at a constant temperature of 25 °C under a 12 h light/dark cycle with free access to food and water. All experiments were approved and performed in accordance with the ethical guidelines of the Institutional Animal Care Committee-CEUA in Federal University of Rio de Janeiro (Approval codes and dates: DFBCICB028 and protocol n° 130/16 [28 March 2017]).

### 4.2. Cecal Ligation and Puncture (CLP) Model

Polymicrobial sepsis was induced by CLP as previously described [28]. In this model, the cecum perforation releases aerobic and anaerobic intestinal bacteria into the peritoneal cavity, causing peritonitis, which progresses to exacerbated inflammation and immune response. Being considered a gold standard model, CLP mimics the human condition in which mice submitted to surgery with fluid resuscitation show an early overwhelming systemic inflammatory response and a hypodynamic state, including a similar cytokine profile production, apoptosis, and tissue damage [65]. By using a CLP model, we developed studies that support host impairment in clinical phenomenon, in which patients who recovered from a severe septic insult are susceptible to long-term consequences triggered by opportunistic pathogens or organ dysfunction [16,28,66].

In briefly, mice were i.p anesthetized with ketamine (112.5 mg/kg; Vetbrands, São Paulo, Brazil) and xylazine (7.5 mg/kg; Vetbrands, São Paulo, Brazil) and a 1 cm midline incision was made on ventral surface of the abdomen. The cecum was exposed, partially ligated below the ileocecal junction, and punctured twice (21G) before returning to the abdominal cavity. Sterile isotonic saline (1 mL) was administered subcutaneously after surgery. Sham-operated mice (Sham) were subjected to an identical laparotomy but without cecal ligation or puncture and were used as controls. All mice were i.p. treated with ertapenem antibiotic (75 mg/kg—Merck Research Laboratory, Whitehouse Station, NJ, USA) at 5, 24, 48, and 72 h post-surgery. The survival rates were determined daily for 15 d. CLP mice exhibited a 30–40% mortality rate after antibiotic treatment.

### 4.3. Subclinical Acute Kidney Injury and ATRvD1 Treatment

SubAKI model was induced in post-septic mice by intraperitoneal BSA injection as previously described [16]. The Sham and sepsis-surviving CLP mice were divided into four groups 15 d after surgery and received 10 g/kg BSA (i.p.) for seven consecutive days. The Sham and CLP groups received 5 μg/kg ATRvD1 (0.1% ethanol, i.v; Cayman Chemicals Co., Ann Arbor, Michigan, USA) at day 15 post-surgery followed by 0.5 μg/kg (0.01% ethanol) 20 min prior to BSA (Sigma-Aldrich, St Louis, Mo, USA) challenge for six days. The control groups received 0.1 or 0.01% ethanol (vehicle, i.v.) at the same days and volumes (100 μL) as the ATRvD1-treated groups. During treatment, the animals were housed in metabolic cages. At day 22, the mice were anesthetized, and blood samples were collected by cardiac puncture. The kidneys were then immediately removed postmortem. One kidney from each animal was used for immunohistological studies while the other one was used for cytokine quantification by enzyme-linked immunosorbent assay (ELISA) and evaluation of the mRNA expression by quantitative reverse transcription polymerase chain reaction (RT-PCR) analysis.

### 4.4. Renal Function Analysis

Mice were housed individually in metabolic cages, and urine was collected, 24 hours prior to euthanasia on day 22, for volume and parameters analysis. The urine samples were centrifuged and stored at −20 °C. The protein and creatinine levels were determined by the pyragollol red method and the alkaline picrate method, using the Gold Analisa Kit 498M and Kit 335, respectively (Gold Analisa Diagnóstica, Belo Horizonte, Brazil).

### 4.5. Kidney Histology

Kidneys were collected, fixed at 4% buffered formalin, embedded in paraffin, and sectioned (5-μm thick). The tissue sections were stained with hematoxylin and eosin and assessed for glomeruli number, glomerular cell number, and interstitial space. Periodic acid Schiff and Picrosirius Red staining were used to evaluate the ECM and collagen deposition, respectively. Data regarding interstitial space, ECM, and collagen deposition were expressed as a percentage of area stained by total tissue area.

### 4.6. Immunohistochemistry Analysis

Kidney sections were immunostained with antibodies (abs) against collagen type I (COL1; cat#sc-28654, Santa Cruz Biotechnology, Santa Cruz, CA) and TGFβ (cat#sc-146, Santa Cruz Biotechnology) at 1:150 dilutions and with abs against collagen type III (COL3; cat#ab7778, Abcam, Cambridge, MA, USA) and fibronectin (cat#ab2413, Abcam) at 1:300 dilutions. As secondary abs, we used HRP-labeled polyclonal anti-rabbit abs (cat#ALI4404, Biosource International, Camarillo, CA, USA) and anti-mouse abs (cat#AMI4404, Biosource International) at 1:1000 dilutions. The antibody reaction products were observed with the chromogen 3,3’-diaminobenzidine tetrachloride (DAB) (Spring Bioscience, Pleasanton, CA, USA). For both kidney histology and immunohistochemistry assay, cortical and medullary areas were photographed with an Olympus BX53 light microscope (Olympus, Tokyo, Japan) and analyzed using Image-Pro Plus software (Media Cybernetics, Rockville, MD, USA). Quantitative analysis of TGFβ positive area was expressed as percentage of area stained by total tissue area.

### 4.7. Immunofluorescence Analysis

Kidney sections were immunolabeled with primary abs against macrophages, using the anti-Iba1 (cat#NCNP24, Fujifilm Wako, Pure Chemical Corporation, Tokyo, Japan) and anti-cleaved casp-3 (cat#9664, Cell Signaling Technology, Danvers, MA, USA) diluted 1:400 in Super Block (cat#AAA125, ScyTek Laboratories, Logan, UT, USA). The slides were incubated with fluorescent secondary Goat anti-rabbit IgG H&L (Alexa Fluor^®^ 488; cat#ab150077, Abcam, Cambridge, MA, USA) diluted 1:400 in blocking solution. The slides were washed and mounted with DAPI-containing mounting medium, covered with glass coverslips, and imaged on a Sight DS-5M-L1 digital camera (Nikon, Melville, NY, USA) connected to an Eclipse 50i light microscope (Nikon) at different magnifications. The images acquired were used for quantifications of Iba1^+^ cells and mean fluorescence intensity (MFI) for cleaved casp-3 in renal tubular cells, using Image J software (NIH).

### 4.8. Cytokine and ECM Protein Gene Expression Analysis by RT-PCR

Kidney specimens were collected, and total RNA was extracted using TRIZOL reagent (Invitrogen, Karlsruhe, Germany) according to the manufacturer’s instructions. The RNA was quantified with NanoDrop™ One (Applied Biosystems, Waltham, MA, USA), and its quality was evaluated by the analysis of nucleic acid purity, and the ratio of the absorbance contributed by the nucleic acid to the absorbance of the contaminants was estimated. All samples showed an absence of contaminants. Complementary DNA (cDNA) synthesis was performed using 1 mg of total RNA as a template and an iScript cDNA Synthesis Kit (Bio-Rad Laboratories, Hercules, CA, USA). Primers targeting the genes for IL-4, IL-10, TGFβ, TNF-α, COL1, COL3, COL4, and fibronectin were designed using Primer Express software version 3.0 (Applied Biosystems, Waltham, MA, USA) and were synthesized by Invitrogen (Table 1). An RT-PCR assay was performed with StepOne™ Plus Real-Time PCR System (Applied Biosystems, Waltham, MA, USA) using Power SYBR Green Master Mix and the relative quantification method using the 2^−ΔΔCt^ calculation. Mouse large ribosomal protein, P0 (RPLP0) gene, was used as an endogenous control. The results were analyzed using StepOne Software v2.3 (Applied Biosystems, Waltham, MA, USA) [67].

### 4.9. ELISA

The right kidney homogenization per animal was performed in 1 mL PBS containing protease inhibitor (Sigma-Aldrich, Switzerland). The homogenates were centrifuged, and the supernatants were obtained. Cytokine levels of chemokine C-C motif ligand 2 (CCL2), IL-1β, IL-4, IL-6, IL-10, matrix metalloproteinase (MMP)-3, MMP-9, TGFβ, and TNF-α were measured using specific ELISA kits (R&D Systems, Minneapolis, MN, USA), according to the manufacturer’s instructions. The results were expressed as pg/mg of total protein.

### 4.10. Statistical Analysis

Differences in the data between groups were analyzed using one-way analysis of variance (ANOVA) followed by Bonferroni post hoc test or a two-tailed unpaired Student’s test. For all analyses, the data were expressed as mean ± SEM. GraphPad Prism 5 Statistical Software Package (GraphPad Software, La Jolla, CA, USA) was used for all statistical analyses. A *p*-value < 0.05 was considered statistically significant.

## 5. Conclusions

To successfully manage the long-term outcomes of sepsis, such as kidney failure, it is essential to identify new effective therapies. Thus, we proposed that ATRvD1 administration attenuates kidney tubulointerstitial damage and negatively modulates inflammatory mediators, cell apoptosis, and cell infiltration intrinsically linked to the fibrosis of sepsis-surviving mice subjected to a SubAKI model.

## Figures and Tables

**Table 1:** Sequence primers used for real-time PCR.

Target	Primer Sequence		Sequence Source
	Forward	Reverse	
IL-4	TTGAACGAGGTCACAGGAGAAG	AGGACGTTTGGCACATCCA	M29854.1
IL-10	GATGCCCCAGGCAGAGAA	CACCCAGGGAATTCAAATGC	NM_010548.2
TNF-α	CACAAGATGCTGGGACAGTGA	TCCTTGATGGTGGTGCATGA	NM_013693.2
TGFβ	GCAGTGGCTGAACCAAGGA	AGCAGTGAGCGCTGAATCG	NM_011577.1
Fibronectin	GTGTAGCACAACTTCCAATTACGAA	GGAATTTCCGCCTCGAGTCT	NM_010233.1
COL1 ^a^	CTTCACCTACAGCACCCTTGTG	TGACTGTCTTGCCCCAAGTTC	NM_007742.3
COL3 ^b^	AAGGCGAATTCAAGGCTGAA	TGTGTTTAGTACAGCCATCCTCTAGAA	NM_009930.2
COL4 ^c^	ACGGGCCAACGCTTCTTC	CATGATCCCAGTCTTTGAGCTCTA	NM_009932.3
RPLP0 ^d^	GCCAGCTCAGAACACTGGTCTA	ATGCCCAAAGCCTGGAAGA	NM_007475.5

^a^ Collagen type I; ^b^ collagen type III; ^c^ collagen type IV; and ^d^ large ribosomal protein, P0.

## Data Availability

The data presented in this study are available from the corresponding author upon request.
